# Supplementary material for: High-resolution mapping demonstrates inhibition of DNA excision repair by transcription factors
Source: eLife. 2022 Mar 15;11:e73943. doi: 10.7554/eLife.73943 (PMC8970589; doi:10.7554/eLife.73943)
Supplement: Figure 5—source data 1. [file elife-73943-fig5-data1.docx]

**Source data for Figure 5D**: Gel shift data showing binding of Reb1 protein to DNA. The first lane (free DNA lane) was not shown in the final figure.
