# Supplementary material for: High-resolution mapping demonstrates inhibition of DNA excision repair by transcription factors
Source: eLife. 2022 Mar 15;11:e73943. doi: 10.7554/eLife.73943 (PMC8970589; doi:10.7554/eLife.73943)
Supplement: Figure 5—source data 2. — Four independent repair gels are included in this source data. [file elife-73943-fig5-data2.docx]

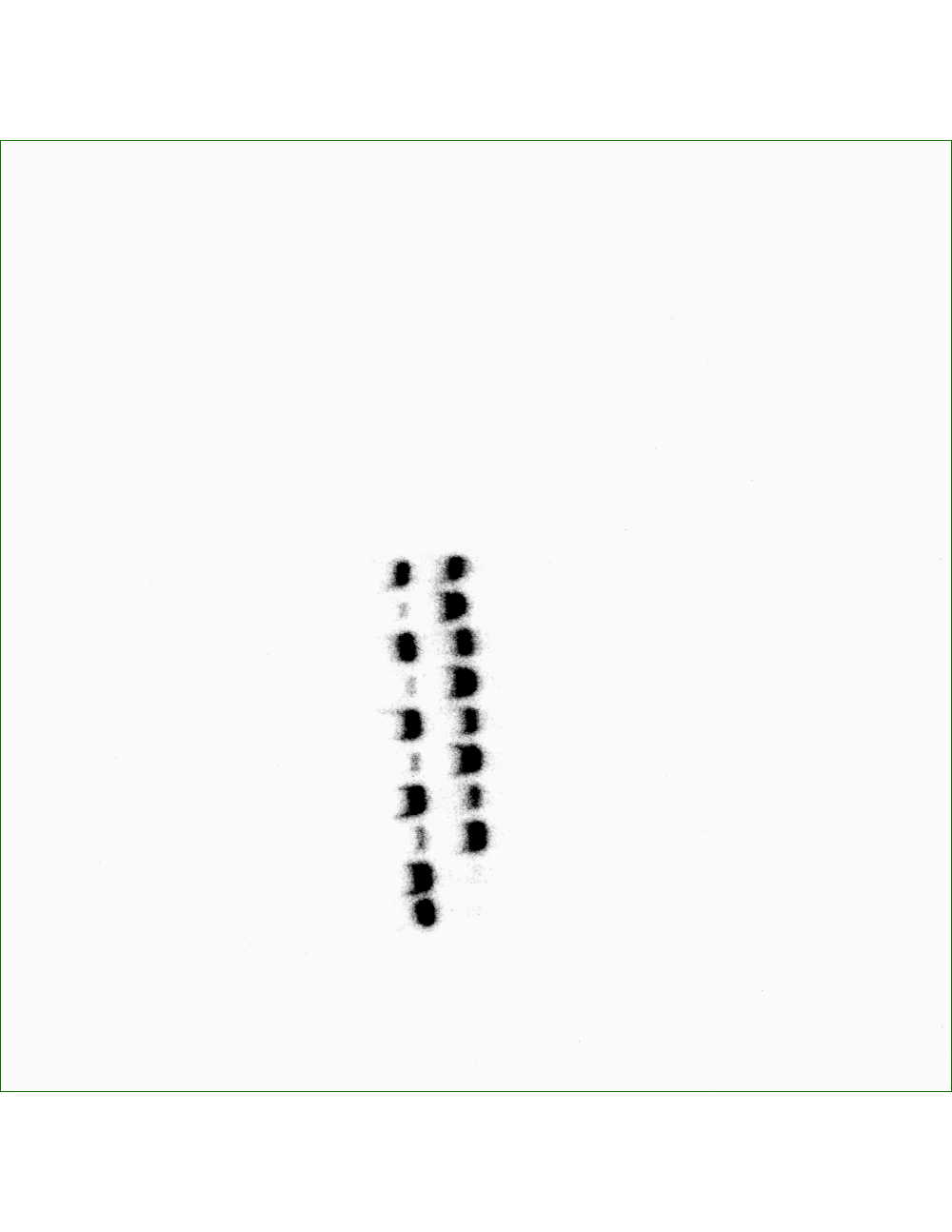


**Source data for Figure 5E**: Cleavage of the inosine-containing DNA or DNA complexed with Reb1 protein. The substrates (naked DNA or DNA-Reb1complex) were incubated with AAG and APE1 enzymes to cleave the damage site. DNA was analyzed on denaturing polyacrylamide gels to separate the full-length DNA (FL DNA) and the cleavage product.


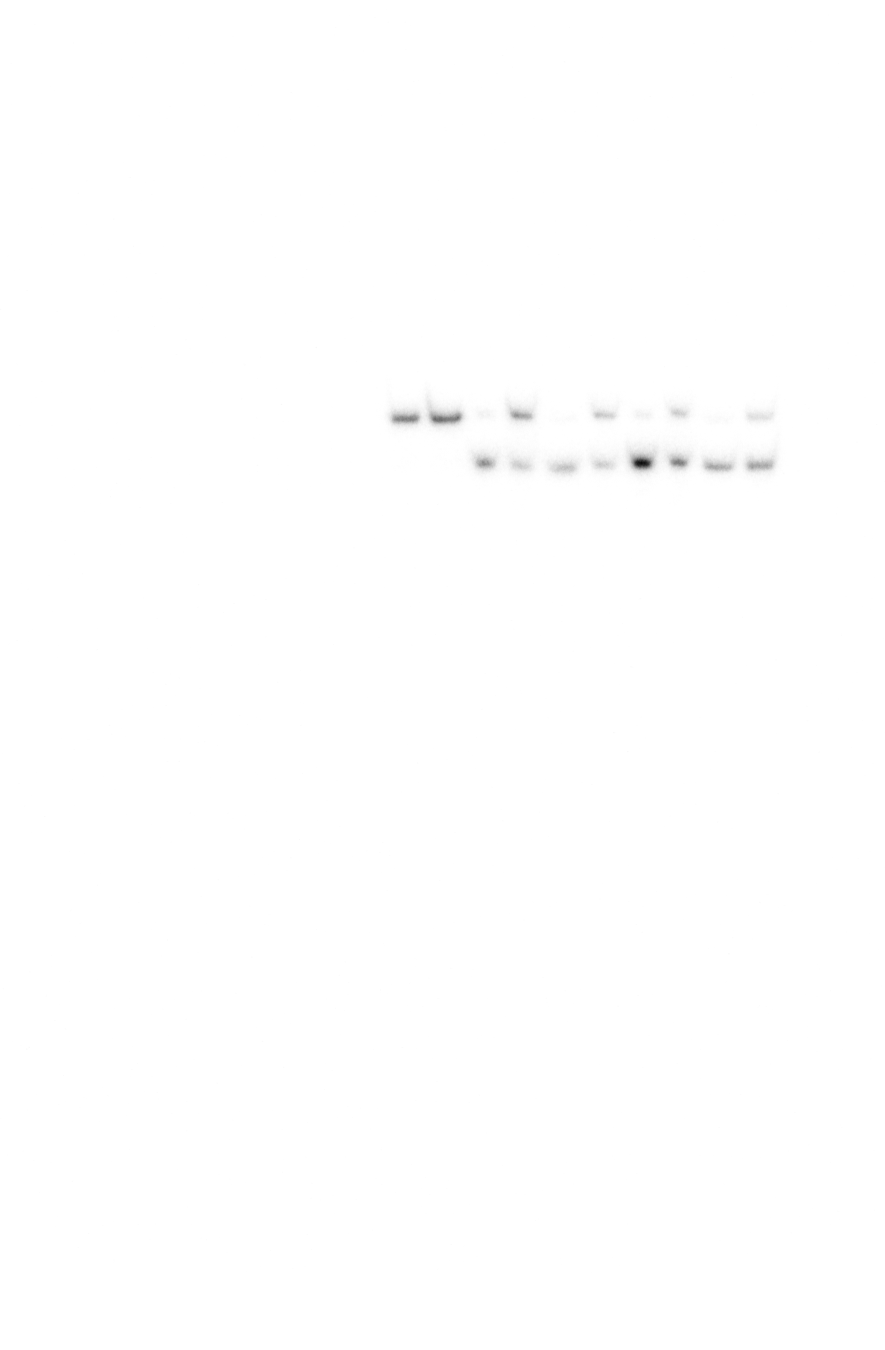


**Source data for Figure 5E**: Cleavage of the inosine-containing DNA or DNA complexed with Reb1 protein. The substrates (naked DNA or DNA-Reb1complex) were incubated with AAG and APE1 enzymes to cleave the damage site. DNA was analyzed on denaturing polyacrylamide gels to separate the full-length DNA (FL DNA) and the cleavage product. This is the 2^nd^ repeat.


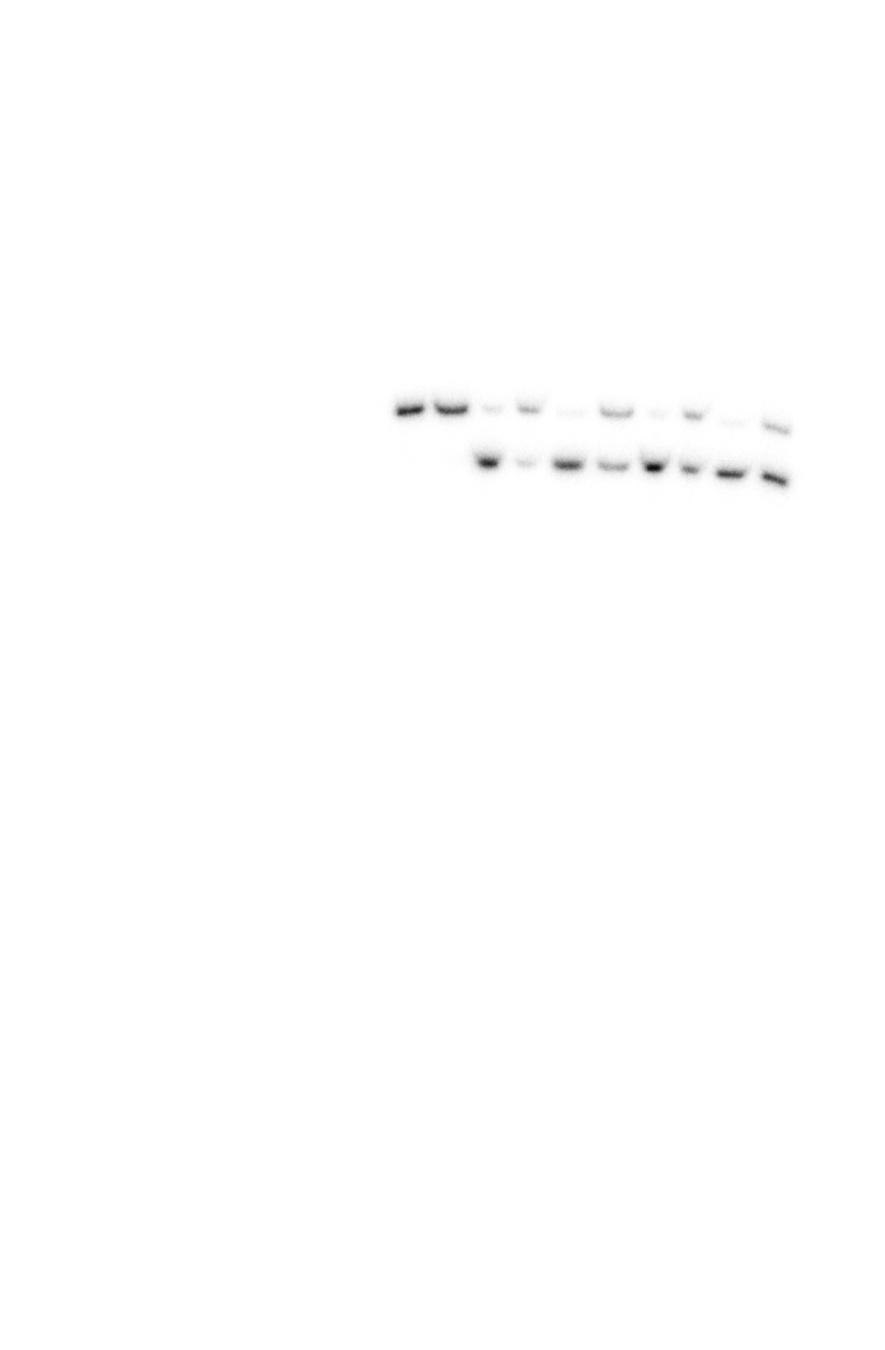


**Source data for Figure 5E**: Cleavage of the inosine-containing DNA or DNA complexed with Reb1 protein. The substrates (naked DNA or DNA-Reb1complex) were incubated with AAG and APE1 enzymes to cleave the damage site. DNA was analyzed on denaturing polyacrylamide gels to separate the full-length DNA (FL DNA) and the cleavage product. This is the 3^rd^ repeat.


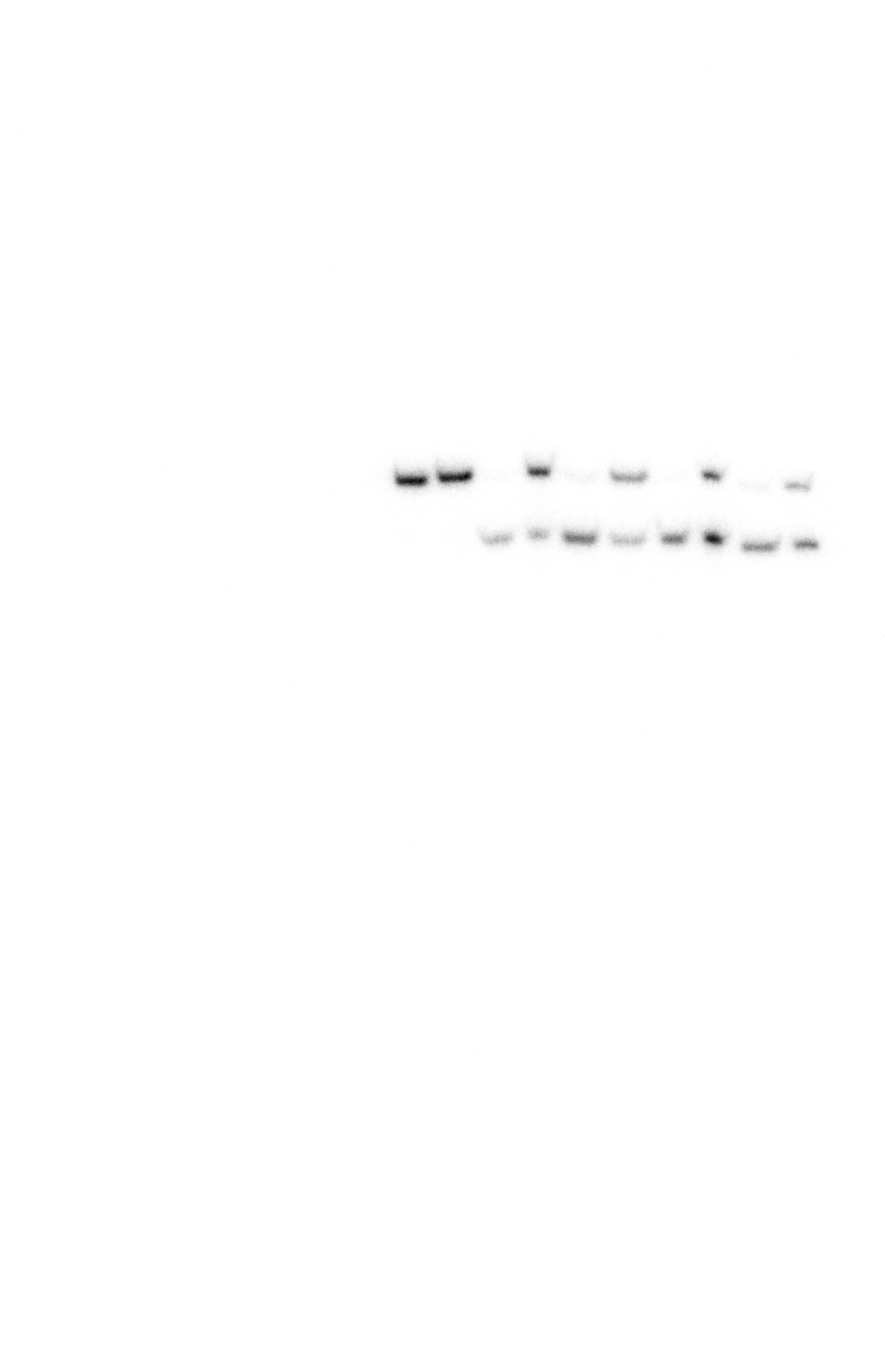


**Source data for Figure 5E**: Cleavage of the inosine-containing DNA or DNA complexed with Reb1 protein. The substrates (naked DNA or DNA-Reb1complex) were incubated with AAG and APE1 enzymes to cleave the damage site. DNA was analyzed on denaturing polyacrylamide gels to separate the full-length DNA (FL DNA) and the cleavage product. This is the forth repeat.
