# Supplementary material for: High-resolution mapping demonstrates inhibition of DNA excision repair by transcription factors
Source: eLife. 2022 Mar 15;11:e73943. doi: 10.7554/eLife.73943 (PMC8970589; doi:10.7554/eLife.73943)
Supplement: Figure 5—source data 3. [file elife-73943-fig5-data3.docx]

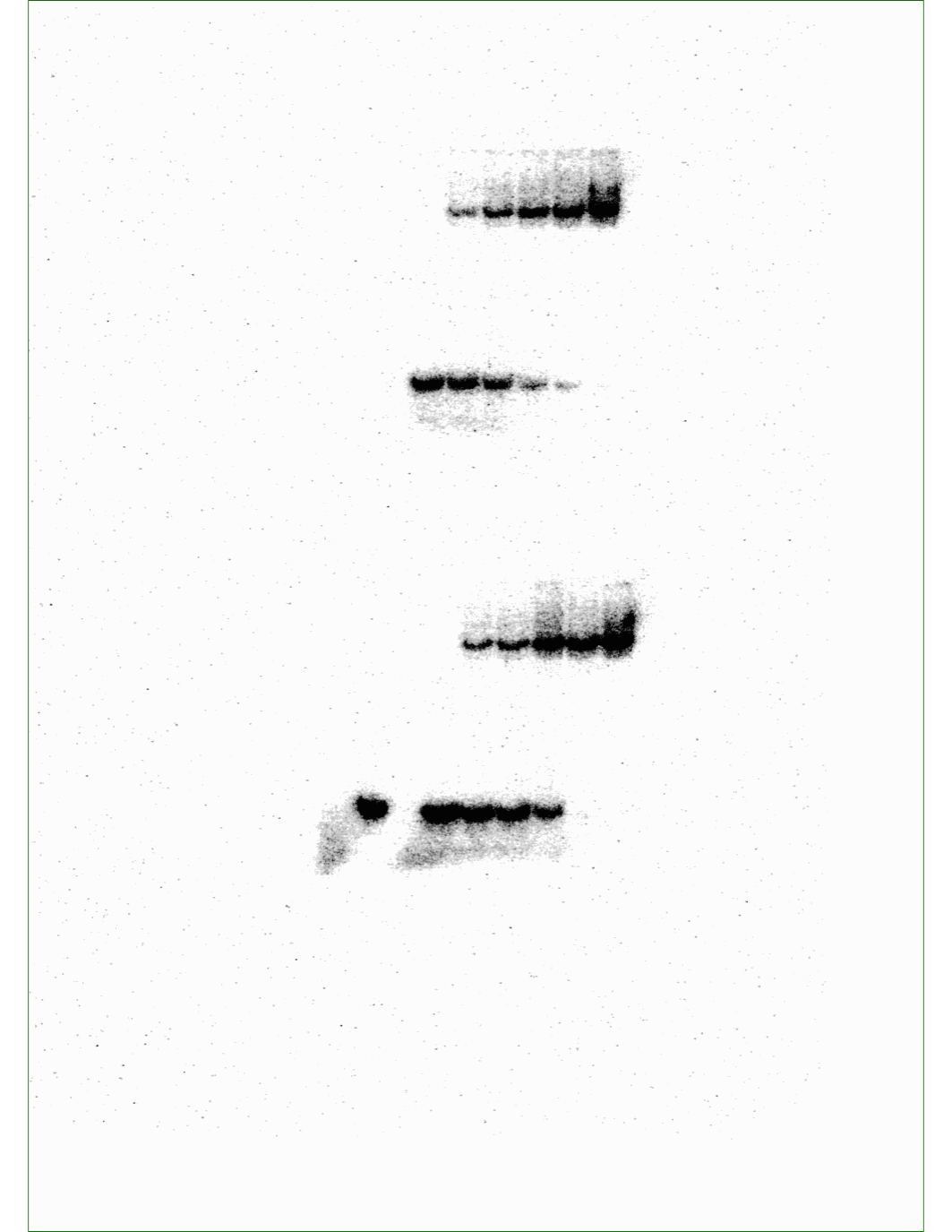


**Source data for Figure 5 — figure supplement 10C**: Gel shift data showing Reb1 binding to undamaged (top) and damaged (bottom) DNA. The bottom gel has an additional free DNA lane on the left, which was not shown in the final figure.
