# Supplementary material for: High-resolution mapping demonstrates inhibition of DNA excision repair by transcription factors
Source: eLife. 2022 Mar 15;11:e73943. doi: 10.7554/eLife.73943 (PMC8970589; doi:10.7554/eLife.73943)
Supplement: Figure 5—source data 4. [file elife-73943-fig5-data4.docx]

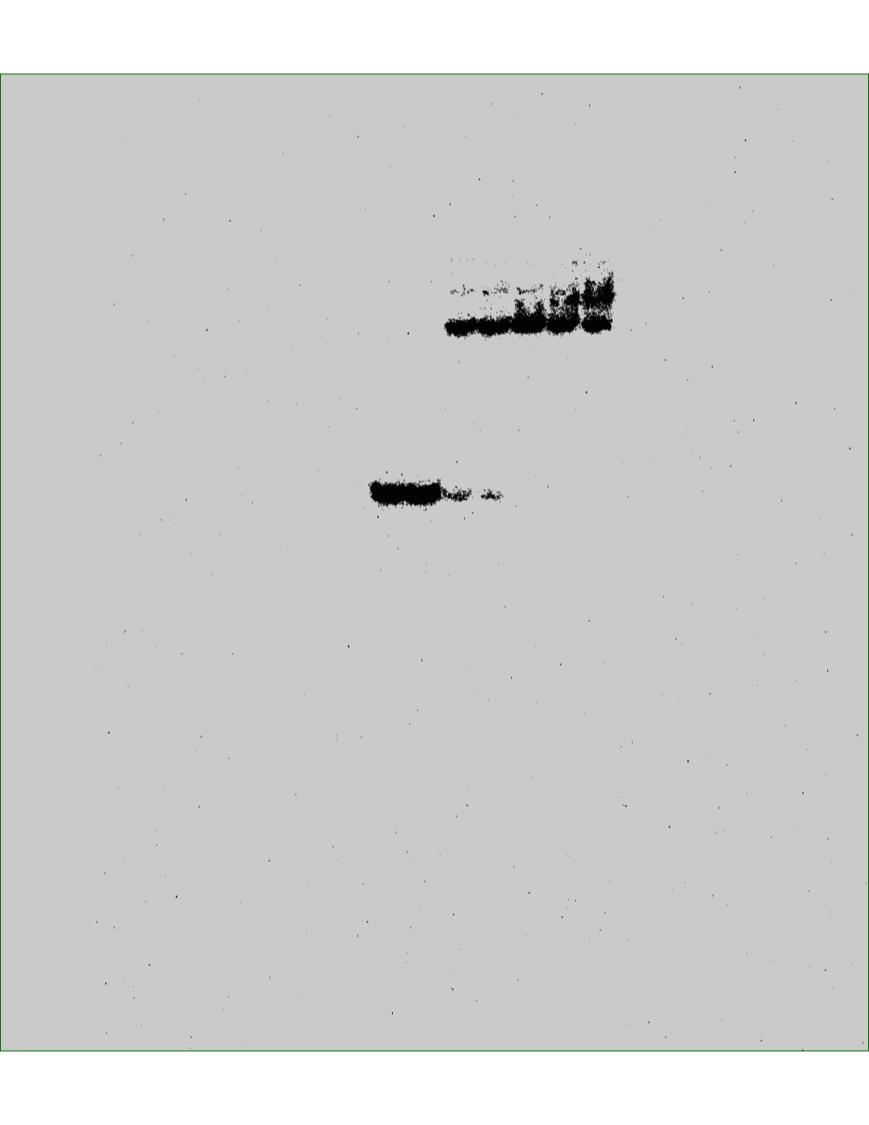


**Source data for Figure 5 — figure supplement 10D**: Gel shift data showing Reb1 binding to DNA (damage is incorporated into the bottom strand). The first two lanes on the left are both free DNA. The first lane was not shown in the final figure.
