# Supplementary material for: High-resolution mapping demonstrates inhibition of DNA excision repair by transcription factors
Source: eLife. 2022 Mar 15;11:e73943. doi: 10.7554/eLife.73943 (PMC8970589; doi:10.7554/eLife.73943)
Supplement: Figure 5—source data 5. — The damage was placed at the +4 position of the non-motif strand. [file elife-73943-fig5-data5.docx]

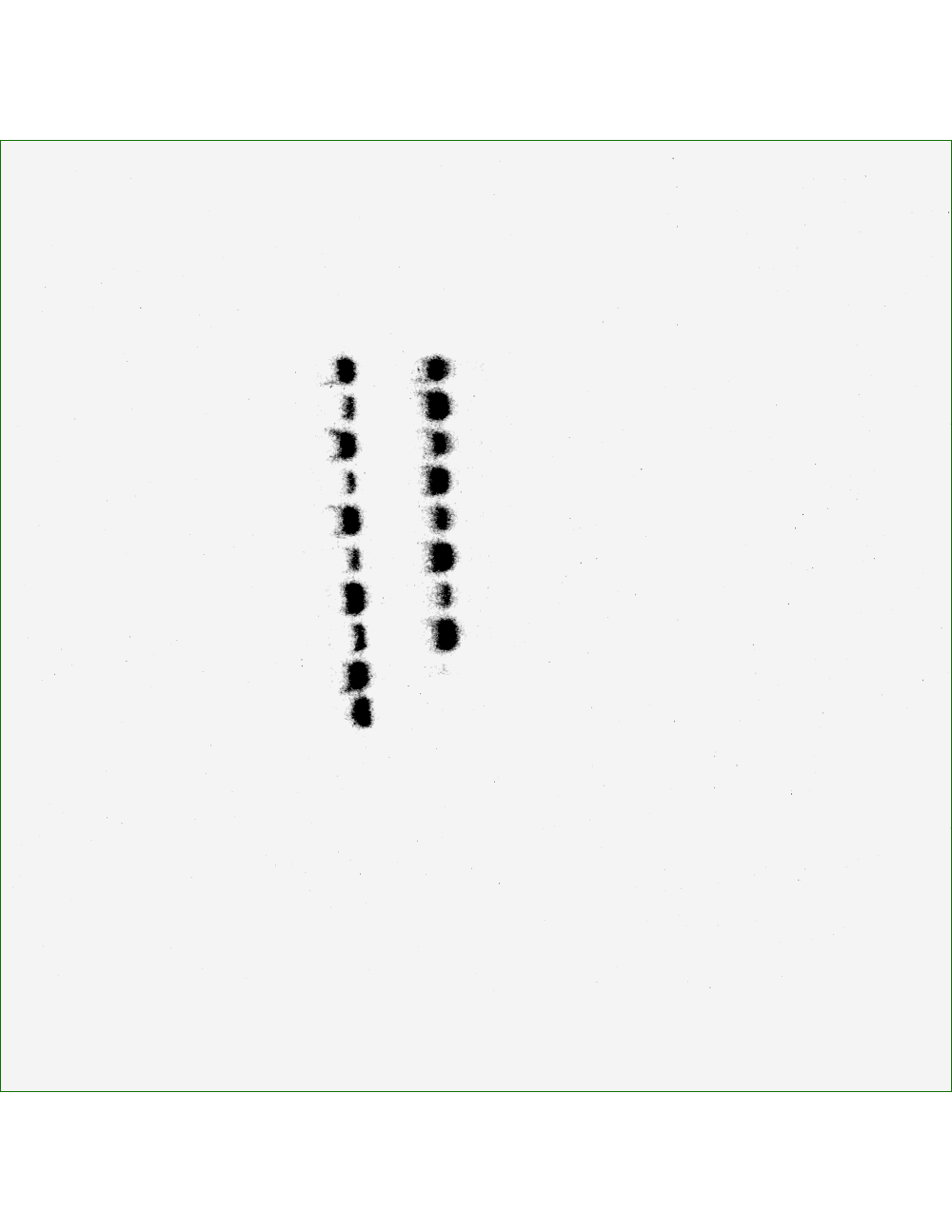


**Source data for Figure 5 — figure supplement 10E**: Cleave of inosine-containing DNA (naked DNA) or DNA bound by Reb1 by AAG/APE1. The damage was placed at the +4 position of the non-motif strand.
